# Qualitative and quantitative analysis of catechin and quercetin in flavonoids extracted from *Rosa roxburghii* Tratt

**DOI:** 10.4314/tjpr.v17i1.11

**Published:** 2018

**Authors:** Ming-Hua Hao, Fan Zhang, Xing-Xia Liu, Fan Zhang, Li-Juan Wang, Sai-Juan Xu, Jin-Hua Zhang, Hong-Long Ji, Ping Xu

**Affiliations:** 1 Department of Pharmacy, Xinxiang Medical University, Xinxiang, Henan, 453003, China; 2 The Third Affiliated Hospital, Xinxiang Medical University, Xinxiang, Henan, 453003, China; 3 Institute of Lung and Molecular Therapy, Xinxiang Medical University, Xinxiang, Henan, 453003, China

**Keywords:** Rosa roxburghii Tratt, Flavonoids, Catechin, Quercetin

## Abstract

**Purpose:**

To perform a qualitative and quantitative analysis of catechin and quercetin in flavonoids extracted from Rosa roxburghii Tratt.

**Methods:**

Total flavonoids were determined using ultraviolet spectrophotometry (UV) at 500 nm. The optimal gradient program started with 15 % methanol and was kept within a period of 0 – 20 min, while 25 % methanol was kept within 20 – 33 min. Subsequently, the concentration of methanol was reduced to 15 % and was held for 10 min until the next injection. Mass spectrometry spray voltage was 4,000 V, ionization temperature 350 °C, atomizer pressure 35 psi, nitrogen flow rate 8 L/min, and mass scan range 200 – 800 m/z. The detection wavelength used for catechin and quercetin was 270 and 368 nm, respectively.

**Results:**

Based on the UV results, Rosa roxburghii Tratt content was 73.85 %, which is in agreement with the national standard. Liquid chromatography-mass spectrometry (LC-MS) results indicate that Rosa roxburghii Tratt flavonoids contained quercetin, 34.26 %, with relative standard deviation (RSD) of 2.88 % and catechin content of 2.97 % with RSD of 1.49 %.

**Conclusion:**

The proposed measurement method for determining the content of flavonoids in Rosa roxburghii Tratt has the advantage of simplicity, feasibility, good repeatability, and rapid and accurate analysis.

## INTRODUCTION

*Rosa roxburghii* Tratt is a Rosaceae deciduous shrub of the genus *Rosa*, and is also known as the silk flower. The nutritional values of *Rosa roxburghii* Tratt are extremely high. Indeed, this fruit is rich in natural antioxidants such as flavonoid compounds, vitamins, and superoxide dismutase [1–4]. In addition, *Rosa roxburghii* Tratt fruit is reported that it has multiple functions such as improving the immune function, anti-atherosclerosis properties, lowering blood pressure, eliminating free radicals, and reducing lipid over-oxidation injury [5–9]. The efficacy of *Rosa roxburghii* Tratt extracts have been evaluated in pancreatic cancer [10], human gastric cancer cells, and human liver cancer cells [11,12]. It was reported that *Rosa roxburghii* Tratt has also beneficial effects on liver cells [13].

*Rosa roxburghii* Tratt contains many flavonoid compounds [5]. Of these, catechin has been reported to exert several beneficial properties, such as scavenging free radicals, anti-mutation properties, radioprotection, inhibition of anaerobic bacteria proliferation, and reduction of high cholesterol [14,15]. Several assays to measure catechin have been reported [16,17]. In addition to catechin, quercetin is another flavonoid of *Rosa roxburghii* Tratt (FRT) and has been reported to lower blood pressure, improve blood capillary resistance, reduce capillary fragility, decrease hematic fat, and increase coronary blood flow [18].

A high performance liquid chromatography (HPLC) method to evaluate catechin and quercetin qualitatively, but no quantitatively, has been reported [19,20]. Therefore, in the present study, we tested the use of a reverse HPLC method to measure both FRTs quantitatively.

## EXPERIMENTAL

### Reagents and equipment

The following reagents were used: *Rosa roxburghii* Tratt (Henan Kaifeng Medical Building, China) and purified catechin and quercetin (Institute for Food and Drug control, China) with at least 98% purity. Furthermore, the following equipment was used: Agilent 1260 HPLC; quaternary pump; diode array detector; auto-sampler; Agilent Chem Station chromatography workstation; Senco R205B rotary evaporator (Shanghai Shen Sheng Technology Co. Ltd, China); Hangping-FA 1104 electronic balance (Shanghai Balance Instrument Factory, China); 202-2-BS electric thermostatic drier (Shanghai Yuejin Medical Instrument Factory, China).

### Extraction and separation of FRTs

*Rosa roxburghii* Tratt was milled into powder and FRTs were extracted using 70 % ethanol at approximately 90 °C, followed by condensing and refluxing for 4 h. The filter residue was discarded, and the filtrate was evaporated using a rotary evaporator. Subsequently, ethanol was removed using the rotary evaporator, and the samples were dried and weighed. A glass packed column served for leak detection. Subsequently, the sample was applied to an HPD 600 macroporous resin-packed column, followed by 4 % HCl and 4 % NaOH activation pillars. After adsorption for 1 h, distilled water was used to remove impurities. Subsequently, desorption was performed by washing with 40 % ethanol eluent, followed by ethanol removing, and sample drying and weighing.

### Determination of total FRTs by ultraviolet (UV) spectrophotometry

Rutin (2.12 mg) was dissolved in methanol and placed in a 10-mL volumetric flask. Subsequently, the dissolved rutin volumes (concentration) of 0 mL (0 %), 0.4 (16.67 %), 0.8 (33.33 %), 1.2 (50 %), 1.6 (66.67 %), 2.0 (83.33 %), and 2.4 (100 %) were transferred to a new 10-mL volumetric flask; the absorbance (A) was subsequently determined. A standard curve was constructed using (A) as the ordinate and rutin concentration (C) as the abscissa. An amount of 19.2 mg of powdered FRT was dissolved in 25 mL of methanol. Subsequently, 0.4 mL of dissolved FRT sample solution was placed into a 10-mL volumetric flask; (A) was determined at 500 nm.

### Determination of quercetin and catechin by HPLC

#### Chromatographic conditions

A Biopearl-HC C18 column (4.6 mm × 200 mm × 5 μm) was used. The mobile phase consisted of methanol (solvent A) and pure water (solvent B), and the flow rate was 1.0 mL/min. The optimal gradient program started with 15 % methanol and was kept in the 0–20 min period, while 25 % methanol was kept in the 20–33 min period. Subsequently, the concentration of methanol was reduced to 15 % and was held for 10 min until the next injection. The column oven temperature was maintained at 30 °C, and the sample injection volume was 20 μL.

### Catechin and quercetin standard reference solution preparation

Twenty-five milligrams of dried catechin and quercetin standard materials were weighed precisely, placed in a 25-mL volumetric flask, and dissolved in methanol at a concentration of 1 mg/mL. The following amounts of standard solutions were placed in a 10-mL volumetric flask: 0.1, 0.2, 0.4, 0.8, 1.6, and 3.2 mL; the volume was adjusted with methanol, and the solutions were mixed by shaking.

A total of 20 μL of the different reference solutions were injected into the drawing precision HPLC peak area as described above in order to measure catechin and quercetin. The standard curve was drawn with the peak area (y) as the ordinate, and the concentration of the reference substance solution (x, μg/mL) as the horizontal coordinate. The regression equations of catechin and quercetin were *y = 8692.6 × – 55.04* (r = 0.9997) and *y = 31738 × – 24.004* (r = 0.9996), respectively. At concentrations of 10–320 μg/mL (r = 0.9997) for catechin and 10–200 μg/mL (r = 0.9996) for quercetin, the two flavonoids showed good linear relationship with the peak area within the scope.

### Determination of quercetin and catechin in the sample solution

An amount of 6.4 mg of powdered FRT was weighed accurately, and subsequently added to 32 mL of methanol and 8 mL of hydrochloric acid to get a final concentration of FRT of 0.2 mol/L. The solution was hydrolyzed in 90 °C for 1 h. Subsequently, the solution was allowed to cool down immediately in ice water for 5 min, transferred to room temperature, and the volume was adjusted up to 25 mL with pure methanol. The solution was filtered using a 0.45-μm organic membrane. An amount of 20 μL was injected to determine the content of the ingredients.

### Precision test

Catechin or quercetin solutions were prepared at 40 μg/mL, and the peak area was determined using 20-μL volumes with five repetitions. The relative standard deviation (RSD) of catechin and quercetin was 1.04 and 0.95 %, respectively, indicating a good measurement precision.

### Repeatability test

The extracted FRTs were divided into five separately prepared solutions. The repeatability analysis was performed as described above to calculate the RSD value of the peak area. The RSD of catechin was 1.83 % (n = 5), while that of quercetin was 1.57 % (n = 5).

### Stability test

A test solution of a known concentration was prepared at room temperature and tested at 0, 2, 4, 6, and 8 h after preparation. Measured according to the chromatographic conditions, the RSD of the catechin content corresponded to 1.4%, while that of quercetin was 1.2 %. The results showed that each test solution was stable within 8 h after preparation.

### Recovery test

A total of 200 μL of the samples were added to 50 μL of a reference solution with a final concentration of 80 μg/mL. The experiment was repeated five times. According to the chromatographic conditions, the recovery results were measured.

### High performance liquid chromatography-mass spectrometry (HPLC-MS) analysis

An Agilent 1260 HPLC Diamonsi IC18 (2.1 mm × 150 mm × 5 μm) was used in the analysis. The mobile phase consisted of methanol (solvent A) and pure water (solvent B), and the flow rate was set at 0.80 mL/min. The optimal gradient program started with 50 % methanol at the time of injection and increased linearly to 70 % methanol over 2 min, which was maintained for 5 min. Subsequently, the gradient was increased linearly to 90 % methanol over 2 min and held for 5 min, followed by a decrease back to 50 % acetonitrile over 0.1 min and held for another 5 min until the next injection. The temperature of the column oven was maintained at 30 °C, and the sample injection volume was 5 μL. The detection wavelengths used for catechin and quercetin were λ_1_ = 270 nm and λ_2_ = 368 nm, respectively.

The MS analysis was performed using the Agilent 1100 series ion trap mass spectrometer with an electrospray ionization source in the positive ion detection mode. The following parameters were used: spray voltage, 4,000 V; ionization temperature, 350 °C; atomizer pressure, 35 psi; nitrogen flow rate, 8 L/min; and mass scan range, 200 – 800 m/z.

### Statistical analysis

The data are expressed as mean ± standard deviation (SD) and were analyzed using analysis of variance (ANOVA) and SPSS/PC (Statistical Package for Social Sciences). Statistical significance was set at *p* < 0.05.

## RESULTS

### Total FRTs

The regression equation of rutin, which was used as the standard, was *y = 10.639 × + 0.0028* (r = 0.9997). At concentrations of 0.01–0.07 mg/mL (r = 0.9997) for rutin, which showed a good linear relationship with the absorbance within the scope, the content of FRTs was 73.85 %; this was in agreement with the national standard.

### Recovery

The catechin and quercetin average recoveries were 98.08 and 98.50 %, respectively, while the RSD values were 1.98 % for catechin and 1.36 % for quercetin. The results of the measurement recovery are summarized in Table 1.

### Qualitative analysis of catechin and quercetin

**Figure 1:**
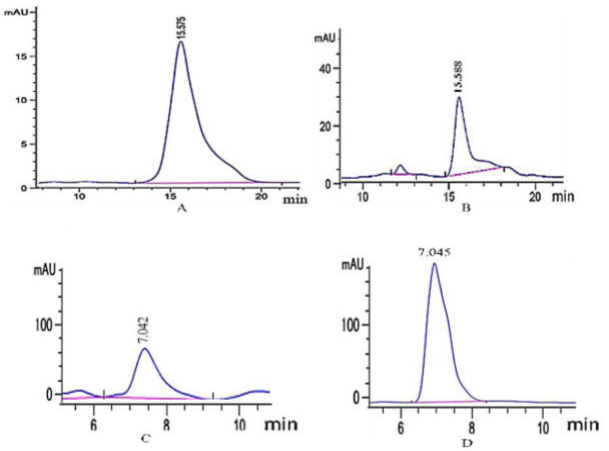
HPLC chromatograms of catechin and quercetin. (A) catechin standard; (B) flavonoid of *Rosa roxburghii* Tratt (FRT) samples; (C) quercetin standard; (D) FRT samples

According to the relevant literature and the mass to charge ratio of the compound, the retention time of catechin standard and FRT samples were comparable (Figure 1); this was also the case for quercetin. As shown in Figure 1, compound 1 contained a fragment of m/z 291.0875, and was consistent with the relative standard molecular weight of catechin (290); thus, compound 1 was catechin. As shown in Figure 1, compound 2 contained a fragment of m/z 303.0425, which is consistent with the relative standard molecular mass of quercetin (302.0); thus, compound 2 was quercetin.

### Contents of catechin and quercetin

The test solution was measured to determine the catechin and quercetin peak areas under the same chromatographic conditions. The measurement was performed in triplicate. Comparison to the standard curve was used to calculate the content of catechin and quercetin, and the results are shown in Table 2.

## DISCUSSION

Flavonoids are polyphenol compounds with 2-phenylchromone as the mother nucleus. These compounds most often exist in a free state or in the form of sugar [21]. Flavonoids provide several beneficial properties, such as radioprotection, anti-free radical, antioxidant, antibacterial, antiviral, anticancer, and cancer prophylactic. In mice, flavonoids have been shown to increase significantly the 30-day survival of radiation and reduce apoptosis and necrosis induced by ionizing radiation [22,23]. Flavonoid protection mechanisms have many advantages, such as DNA protection, antioxidative effects, protection of the immune system, protection of the hematopoietic system, and mitigation of inflammation. The *Rosa roxburghii* Tratt bioactivity can be studied not only based on its flavonoids but also on its radioprotective activity of isolated monomeric compounds. It also has a good application prospect to compare the direct activity difference of different monomer components [24]. The ratio of methanol and water is very important when measuring isolated flavonoids. In this study, this approach was evaluated using various conditions including methanol-water (10:90), acetonitrile-water (10:90), methanol-0.1 % phosphoric acid buffer solution (10:90), and other mobile phases. Eventually, the gradient elution conditions started with 15 % methanol at the time of injection, increased linearly up to 25 % for 13 min, and decreased subsequently back to 15 % methanol, which was held for 10 min until the next injection. The advantage of this condition was that it could separate catechin and quercetin and had better peak shapes. Dual wavelength determination was used to measure catechin and quercetin at 368 nm and 270 nm, respectively, which allowed maximum absorption, improved the resolution, and smoothed the baseline.

## CONCLUSION

Accurate measurement of the levels of catechin and quercetin flavonoids in the fruit of *Rosa roxburghii* Tratt is important for further investigation of their benefits. The experimental determination of these two flavonoids may provide a theoretical basis and quality assessment for the rational development and utilization of *Rosa roxburghii* Tratt.

## Figures and Tables

**Figure 2:**
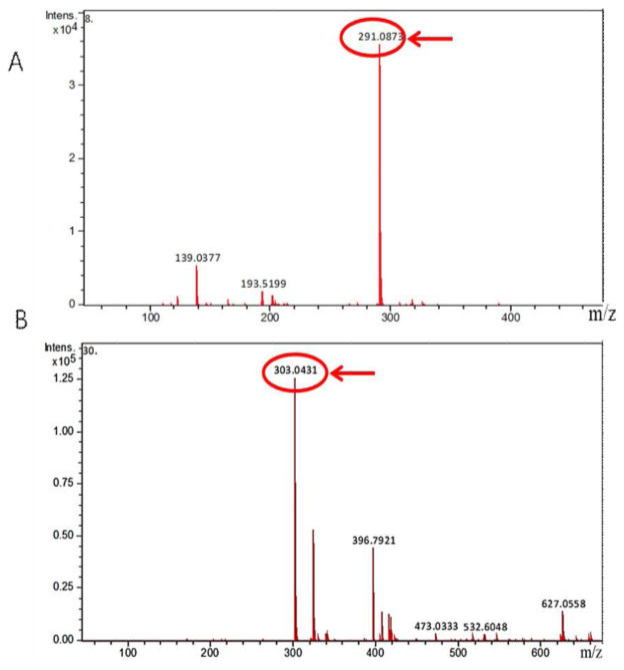
Mass spectra of compounds in flavonoid of *Rosa roxburghii* Tratt (FRT). (A) Mass spectrum of catechin in FRT; (B) mass spectrum of quercetin in FRT

**Table 1:** Recovery data

Compound	Original content (μg/mL)	Addition (μg/mL)	Measured value (μg/mL)	Recovery (%)	Average recovery (%)	RSD (%)
Catechin	110.45	20	130.06	98.05	98.08	1.98
	110.45	20	129.95	97.48		
	110.45	20	130.28	99.15		
	112.76	20	131.81	95.25		
	112.76	20	132.85	100.45		
Quercetin	20.45	20	40.12	98.35	98.05	1.36
	20.45	20	40.38	99.65		
	20.45	20	40.46	100.04		
	21.63	20	41.13	97.50		
	21.63	20	41.02	96.95		

RSD = relative standard deviation

**Table 2:** Contents of catechin and quercetin (n = 3)

Sample	Content (%)	*X̄*	RSD (%)
Catechin	37.26	34.26	2.88
	34.01		
	31.51		
Quercetin	2.65	2.97	1.49
	3.16		
	3.10		
